# Enrollment of dengue patients in a prospective cohort study in Umphang District, Thailand, during the COVID‐19 pandemic: Implications for research and policy

**DOI:** 10.1002/hsr2.1657

**Published:** 2023-11-08

**Authors:** Donald S. Shepard, Priya Agarwal‐Harding, Sukhum Jiamton, Eduardo A. Undurraga, Sukhontha Kongsin

**Affiliations:** ^1^ Schneider Institutes for Health Policy Heller School for Social Policy and Management, Brandeis University Waltham Massachusetts USA; ^2^ Research Centre for Health Economics and Evaluation, Faculty of Public Health Mahidol University Bangkok Thailand; ^3^ Department of Dermatology, Faculty of Medicine Siriraj Hospital Mahidol University Bangkok Thailand; ^4^ Escuela de Gobierno Pontificia Universidad Católica de Chile Santiago Chile; ^5^ Research Center for Integrated Disaster Risk Management (CIGIDEN) Santiago Chile; ^6^ CIFAR Azrieli Global Scholars Program Toronto Ontario Canada

**Keywords:** cohort, dengue, district hospital, global health, neglected diseases, Thailand

## Abstract

**Background and Aims:**

Dengue is endemic in Thailand and imposes a high burden on the health system and society. We conducted a prospective cohort study in Umphang District, Tak Province, Thailand, to investigate the share of dengue cases with long symptoms and their duration. Here we present the results of the enrollment process during the COVID‐19 pandemic with implications and challenges for research and policy.

**Methods:**

In a prospective cohort study conducted in Umphang District, Thailand, we examined the prevalence of persistent symptoms in dengue cases. Clinically diagnosed cases were offered free laboratory testing, We enrolled ambulatory dengue patients regardless of age who were confirmed through a highly sensitive laboratory strategy (positive NS1 and/or IgM), agreed to follow‐up visits, and gave informed consent. We used multivariate logistic regressions to assess the probability of clinical dengue being laboratory confirmed. To determine the factors associated with study enrollment, we analyzed the relationship of patient characteristics and month of screening to the likelihood of participation. To identify underrepresented groups, we compared the enrolled cohort to external data sources.

**Results:**

The 150 clinical cases ranged from 1 to 85 years old. Most clinical cases (78%) were confirmed by a positive laboratory test, but only 19% of those confirmed enrolled in the cohort study. Women, who were half as likely to enroll as men, were underrepresented in the cohort.

**Conclusions:**

The Thai physicians' clinical diagnoses at this rural district hospital had good agreement with laboratory diagnoses. By identifying underrepresented groups and disparities, future studies can ensure the creation of statistically representative cohorts to maximize their scientific value. This involves recruiting and retaining underrepresented groups in health research, such as women in this study. Promising strategies for meaningful inclusion include multi‐site enrollment, offering in‐home or virtual services, and providing in‐kind benefits like childcare for underrepresented groups.

## Key points

- Cohort studies are a powerful tool for examining correlates of major diseases, such as dengue
- The validity of the resulting data, however, depends on the representativeness of the underlying cohort
- During the COVID‐19 pandemic, only a small share of eligible patients were willing to join the cohort study in Thailand's Tak Province
- Comparisons with external data showed that adult women were particularly underrepresented in the cohort due to their household and childcare responsibilities
- The analyses showed how cohort studies could be improved by identifying underrepresented groups, offering targeted support services and performing statistical weighting

## 1 INTRODUCTION

The incidence of dengue continues to grow, with estimates of about 100 million symptomatic infections yearly in over 100 countries.^1^, ^2^, ^3^ Dengue is endemic in most tropical and subtropical regions, imposing a severe disease and economic burden.^4^ Southeast Asia has the world's highest dengue incidence, with all four dengue virus (DENV) serotypes found in most countries.^5^, ^6^ Dengue is considered a major public health threat in Thailand and is a notifiable disease (Supporting Information: Section S1).^7^, ^8^, ^9^, ^10^, ^11^, ^12^, ^13^

Critical knowledge gaps about dengue remain.^14^, ^15^ Most existing studies are urban, focus on hospitalized patients, cover only the acute phase of dengue illness, include children primarily, are time‐limited, and employ only crude impact measures. Notably, longitudinal prospective cohorts improve our understanding of dengue epidemiology, including the natural course of the disease and its long‐term impacts on individuals and populations, and strengthen the public health response.^16^ However, the quality and reliability of scientific evidence from these studies depend on participants’ enrollment. Enrollment is a critical step in the design of longitudinal cohort studies, as it determines the sample size and the representativeness of the study population. If enrollment is selective, the sample may not accurately represent the population being studied, leading to biased or unreliable results. This, in turn, limits the generalizability of the findings and can compromise the optimal design of public health policies and interventions.

We conducted a longitudinal prospective cohort study in Thailand to investigate the share of dengue cases with persistent symptoms. Here we present the results of the enrollment process during the COVID‐19 pandemic, highlighting some critical challenges and implications of conducting longitudinal cohort studies during a public health crisis. Our study builds on previous dengue research in Thailand.^7^, ^8^, ^9^, ^13^, ^17^, ^18^, ^19^, ^20^ It provides insights to improve the design of dengue cohorts that could inform clinical, epidemiological, and economic models for dengue control interventions.

## 2 MATERIALS AND METHODS

### 2.1 Study population and design

**Figure 1:**
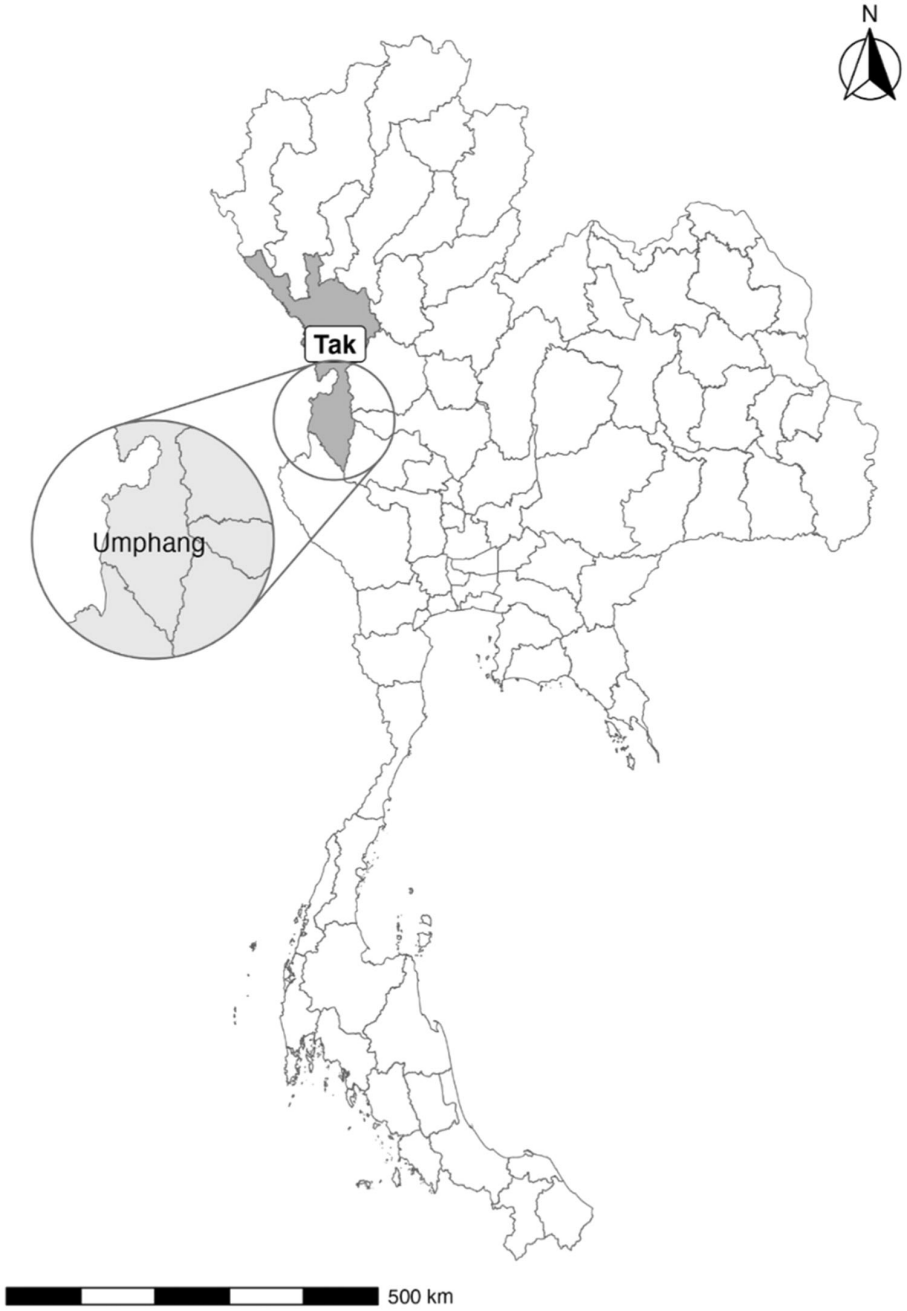
Map of study site in Umphang District (UMP), Tak Province, Thailand.

The study was implemented in Umphang District, a rural district with the highest dengue incidence rate in the dengue‐endemic province of Tak, Thailand (Figure 1). Based on the latest official surveillance data,^21^ the dengue incidence in Tak Province slightly exceeded Thailand's national average in Thailand. In 2020, Tak Province reported an incidence rate of 190 cases compared to the country's average of 187 cases per 100,000 population. In Tak Province and Thailand overall, the rates of dengue hemorrhagic fever were 56 and 60, and the rates for dengue deaths were 0.00 and 0.02 per 100,000 population, respectively.

We defined a suspected dengue episode as an apparent DENV infection in which the infected person visits a clinic or hospital seeking professional healthcare and DENV infection was clinically diagnosed by medical doctors. Clinical diagnoses are based on epidemiological reasoning, symptoms (e.g., headache, fever, nausea, and bleeding diathesis), a tourniquet test, and hematocrit, platelets, and hematological parameters, following WHO technical guidelines.^22^

A global panel of health experts has rated Thailand's health system as one of the five best in low‐ and middle‐income countries. One of Thailand's strengths was the adequate staffing of doctors and nurses in rural district health services, such as Umphang, thanks to expanded training positions and a 3‐year posting requirement for doctors. Other strengths were the country's near universal (97.7%) health insurance coverage, its meager (only 16.5%) share of out‐of‐pocket payments for health care, and its innovative response to crises, such as the COVID‐19 pandemic.^23^, ^24^ As a result, most patients in the catchment area with suspected dengue likely came to the health system for diagnosis and treatment, making the screened cohort relatively complete.

During the enrollment phase, the study offered free laboratory testing in the Umphang Hospital, consisting of the NS1 (nonstructural protein 1 antigen) rapid dengue test and rapid Immunoglobulins G and M (IgG/IgM) tests to suspected dengue patients. Due to cost and availability constraints, these tests are not routinely undertaken for suspected dengue patients in Thailand. Patients who initially tested negative on both tests (which may occur based on the time elapsed since DENV infection or whether the infection is the first or second^25^) could request a repeat IgG/IgM test at their next visit. Eligibility for the study had no age limitations but required that the patient had laboratory‐confirmed dengue by testing positive for either Ns1 or IgM and was available for follow‐up visits (Supporting Information: Section S2). We recorded about 25 variables on each patient related to demographics and screening. Participants were compensated 500 Baht (approximately US$16) for each visit. All enrollees were ambulatory patients.

### 2.2 Data collection and training

Training and tools for interviewing, data entry, data cleaning, and verification were provided in two field visits to Umphang Hospital in 2020 and 2022. We used Microsoft Access (Redmond, WA) for double‐entry verification. The Thai Department of Provincial Administration collected population counts by age group.

### 2.3 Data analysis

As our prespecified goal was to enroll a representative cohort of laboratory‐confirmed dengue patients, analyses within our cohort were all exploratory. We calculated descriptive statistics of the patients screened and enrolled in our study. We derived each age group's share of clinically and laboratory‐confirmed dengue cases screened in our study relative to its share in the overall population of Thailand by age group. We assessed the associations among screening frequencies, patient characteristics, and month of screening using Pearson's correlations and Chi‐square tests (*X* ^*2*^). We modeled the probability of laboratory confirmation of clinical dengue using multivariate logistic regressions for NS1 alone, IgM alone, and any laboratory confirmation test (NS1 and/or IgM) as dependent variables and the days elapsed since the onset of illness as covariates. Lastly, we tested whether patients’ characteristics (gender, age), month of screening, and days elapsed from the onset of symptoms to hospital presentation were significantly associated with enrollment into our study (defined as being below or equal to a *p* < 0.05 level) using Chi‐square tests or two‐way Fisher's exact tests. Significance tests were all two‐sided. All analysis was carried out using STATA 17 statistical software.

### 2.4 Ethics approval

All adult participants (age 18 and above) gave written informed consent. For all child participants (under age 18), the child's legal caretaker gave written informed consent. In addition, child participants aged 15–17 gave written assent. The ethical committees at Tak Provincial Health Department, Mahidol University, and Brandeis University reviewed and approved the study. The study team was responsible for the study design, data collection, and analysis.

### 2.5 Study integrity

All authors have read and approved the final version of the manuscript. The corresponding author (DSS) had full access to all of the data in this study and takes complete responsibility for the integrity of the data and the accuracy of the data analysis.

## 3 RESULTS

### 3.1 Patients screened

We recruited 150 patients for screening from December 2020 through November 2021. Most cases occurred during the high dengue season (May 1 through August 31, 2021), with the peak in June (39%, *N* = 59) (Supporting Information: Figures S1–S3). Laboratory confirmation for dengue with NS1 was significantly more likely during the high dengue season compared to the rest of the year (*X* ^*2*^[1, *N* = 150] = 17.65, *p* < 0.001). Month was a significant predictor of a positive NS1 test *X* ^*2*^(8, *N* = 150) = 22.77, *p* = 0.004. Any laboratory confirmation (NS1 and/or IgM) tended to be more likely during the high dengue season (*X* ^*2*^[1, *N* = 150] = 3.68, *p* = 0.06) and month tended to be a predictor of any laboratory confirmation (*X* ^*2*^[8, *N* = 150] = 15.26, *p* = 0.05). Of the patients who were screened and tested (*N* = 150, Supporting Information: Table S1), 78 patients (52%) tested positive for NS1, while 90 patients (60%) tested positive for IgM and/or IgG, which indicates either a past or current dengue infection. Altogether, 117 (78%) of the 150 clinical dengue cases were lab confirmed for a current infection (tested positive for NS1 and/or IgM) and 30 (20%) were positive for both tests. Including IgG, 138 (92%) patients were positive on any dengue test (NS1, IgG, or IgM), resulting in almost all clinically diagnosed patients having lab confirmation of current or past dengue.

**Figure 2:**
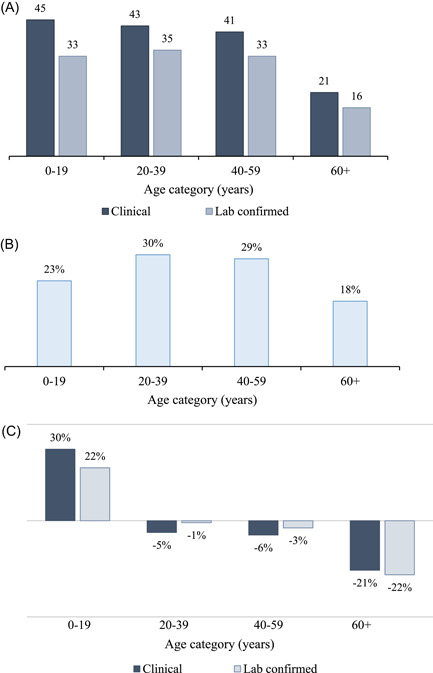
Age distribution of suspected and confirmed dengue cases. The panels show the (a) number of clinical and lab‐confirmed dengue cases by age group, (b) population distribution in Thailand (percentage of the population in each age group), and (c) difference in the incidence of dengue cases in the study population relative to the population of Thailand by age group.

Table 1 shows the characteristics of the patients screened and enrolled. We enrolled 22 patients (all outpatients) who tested positive, were eligible, and consented from April 5th through July 1st, 2021. Diagnoses included dengue hemorrhagic fever (DHF, *n* = 3) and dengue fever (DF, *n* = 19). The age range was 1–88 years (average 35) in those screened and 7–76 years (median 34) in enrollees. The age distributions of both suspected and confirmed cases (Figure 2) suggest that the group under 19 years had a higher incidence of dengue (was overrepresented) relative to the population of Thailand. The oldest group (age 60+) was underrepresented.

**Table 1:** Number of suspected dengue patients screened, laboratory confirmed, and enrolled in the cohort study in Umphang Hospital, Tak Province, Thailand, from December 2020 through November 2021.

Item	Clinical cases	Screened (%)	Lab‐confirmed	Laboratory conf. rate	Enrolled	Enrollment rate	Weighting factor	Enrollment relat. rate
Total	150	100	117	78%	22	19%	1.00	1.00
Screening months								
Dec^†^–Jan	9	6	7	78%	0	0%	n.a.	0.00
Feb–Mar	4	3	3	75%	0	0%	n.a.	0.00
Apr–May	41	27	29	71%	14	48%	0.39	2.57
Jun–Jul	94	63	76	81%	8	11%	1.79	0.56
Aug–Sep	2	1	2	100%	0	0%	n.a.	0.00
Oct–Nov	0	0	0	n.a.	0	n.a.	n.a.	n.a.
Gender								
Male	82	55	66	80%	16	24%	0.78	1.29
Female	68	45	51	75%	6	12%	1.60	0.63
Age (years)								
0–19	45	30	33	73%	5	15%	1.24	0.81
20–39	43	29	35	81%	7	20%	0.94	1.06
40–59	41	27	33	80%	7	21%	0.89	1.13
60+	21	14	16	39%	3	19%	1.00	1.00
Elapsed days								
≤0	6	4	6	100%	0	0%	n.a.	0.00
1–3	93	62	76	82%	1	1%	14.29	0.07
4–6	41	27	29	71%	18	62%	0.30	3.30
7–9	9	6	5	56%	3	60%	0.31	3.19
10–12	0	0	0	n.a.	0	n.a.	n.a.	n.a.
≥13	1	1	1	100%	0	0%	n.a.	0.00

*Notes:* ^†^ December 2020. Conf.: confirmation. Relat.: relative. Patients were considered laboratory‐confirmed for dengue if they were positive for NS1 and/or IgM. The lab confirmation rate is the number of positives as a percentage of the number of persons screened in that row. The enrollment rate is the number of patients enrolled as a percentage of the number of persons positive in that row. The enrollment weighting factor, calculated as the overall enrollment rate (19%) divided by the enrollment rate in that row, is the weight that could be applied to participants in that category to make them representative of all lab‐confirmed patients. The enrollment weighting factor cannot be estimated for rows with no enrollees. The enrollment relative rate is the enrollment rate for that row divided by the overall enrollment rate.

We found an average of 2.9 days (SD = 4.29) between the self‐reported onset of illness and presentation to the hospital for screening across the study period. Only three patients reported no symptoms before presenting to Umphang Hospital; 120 patients (80%) took between 1 and 4 days from the onset of symptoms to seek healthcare. Lastly, 24 patients (16%) waited more than 4 days from the onset of symptoms to seeking healthcare; 10 patients (7%) waited more than 1 week. In peak season (May–September), there was an average of 3.04 days (SD = 3.69) between the onset of illness and screening (hospital presentation), compared to 2.38 days (SD = 7.06) during the non‐peak season.

### 3.2 Patients lab confirmed

**Figure 3:**
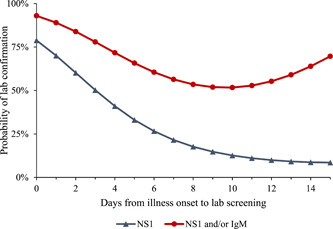
Modeled probability of laboratory confirmation for suspected dengue. Probabilities are estimated based on quadratic logistic regressions. The logistic regressions exclude three observations with negative values for days between illness onset and hospital presentation, so they are based on the remaining 147 observations. Both regressions were statistically significant (*p* = 0.0007 for NS1 and *p* = 0.0164 for NS1 and/or IgM). However, the regression for IgM alone was insignificant (*p* = 0.24) and was not used.

Figure 3 shows the modeled probability of laboratory confirmation for suspected dengue episodes using quadratic logistic regressions with NS1, IgM, and NS1 and/or IgM as the dependent variables and days elapsed since the onset of illness as covariates (Supporting Information: Figure S4 shows raw data). Both regressions were statistically significant (*p* < 0.001 for NS1 and *p* = 0.02 for NS1 and/or IgM). However, the regression for IgM alone was not significant (*p* = 0.24), so we excluded this result from Figure 3.

Most enrollees were men (*n* = 16/22, 73%) (Supporting Information: Figure S5). However, the relationship between gender and enrollment rate was borderline significant at *p* = 0.10. Most patients were enrolled in May 2022 (*n* = 13, 59%); we found a significant correlation between the month of screening and enrollment *X* ^*2*^(8, *N* = 150) = 21.57, *p* = 0.006. We found no significant association between patients' age or days elapsed from the onset of symptoms to hospital presentation with enrollment into our study.

## 4 DISCUSSION

The enrollment process in a prospective cohort study in Umphang District during the COVID‐19 pandemic showed several noteworthy results related to diagnostic accuracy, enrollment, and age patterns. Our study revealed a remarkably high proportion of laboratory‐confirmed DENV infections (78%, *N* = 117/150) among clinically diagnosed patients, providing evidence of the accuracy of clinical diagnoses in the cohort. Plausible explanations for this high accuracy rate include the clinicians' extensive experience with dengue, utilization of epidemiological reasoning and comprehensive clinical signs and symptoms, including a tourniquet test, and access to hematological lab tests and incorporation of relevant hematological parameters in the diagnosis of patients, including elevated hemoglobin, hematocrit, and monocyte count, and lower white blood cell and platelet counts.^26^, ^27^ These clinical tests may not be routinely done in all hospitals in Thailand but depend on the director's judgment or resource availability. However, our findings support using these criteria for case diagnosis and reporting, especially in the absence of laboratory tests and antiretrovirals.

Another reason for the high rate of laboratory confirmation of clinical diagnosis was the combination of NS1 and IgM that captured dengue cases with both recent and earlier onset. In contrast, a clinical trial with Thai control‐arm participants showed that only 12.1% of fever episodes alone (but not necessarily a clinical dengue diagnosis) were virologically confirmed dengue.^28^ Data accuracy extended to patients, who were conscientious in completing lab tests and providing requested data.

Laboratory testing within a dengue cohort is valuable, serving multiple purposes and offering valuable insights. In terms of clinical management, the inclusion of testing greatly enables the confirmation of dengue virus (DENV) infection through the detection of dengue‐specific antigens (e.g., NS1) or antibodies (e.g., IgM). This distinction is vital for accurately differentiating dengue cases from other febrile illnesses and informing treatment according to the stage of the disease. Additionally, it is critical that researchers and public health practitioners accurately estimate the incidence of dengue cases to best design and roll out interventions, including vaccinations. For each of these purposes, it is important to note that a tradeoff exists between sensitivity and specificity in testing. Our utilization of NS1 testing exhibited high specificity, guaranteeing that all participants within the cohort had dengue. However, it may have missed some cases due to its lower sensitivity.

Enrollment was a challenge in our study; only 22 patients (19% of lab‐confirmed patients) enrolled. This may be partly explained due to a decline in reported DENV cases in Tak province from 1214 and 1337 in 2018 and 2019 to 754 and 355 in 2020 and 2021, respectively.^21^ Thailand as a whole also saw substantial declines in numbers of reported dengue cases from 2019 to 2020 and again to 2021.^29^ Reasons included higher immunity following peak rates in 2019, less rainfall, and reduced travel and exposure due to COVID‐19 restrictions. Of all the 2‐month periods, June/July showed the highest number of cases screened (94), with the next largest (April/May) only 41. The timing of this peak period is consistent with Thailand's epidemiological data for prior years.^29^

The COVID‐19 pandemic compounded the decline in dengue incidence with low enrollment rates. Study recruiters reported that several lab‐confirmed patients refused to enroll due to concerns of contagion related to the COVID‐19 outbreak and the long distances in public transport and the number of hospital visits that enrollment in the study would have required. Travel was a common concern, particularly among women, resulting in a 2.06 underrepresentation of females compared to men. Women's greater childcare responsibilities and difficulties finding transportation may have factored into this difference. However, once patients committed to enrolling, they were highly adherent in completing the expected tests and furnishing the requested data. During COVID, when patients were reluctant to travel and use communal transportation, several potential enrollees reported inadequate compensation.

Gender imbalance is persistent in many cohort studies,^30^, ^31^ including those conducted in urban areas. Two potential solutions to facilitate participation, where feasible, are to utilize home visits or telehealth technology. Participants who may have difficulty traveling to study sites or attending in‐person appointments may then be more likely to enroll and remain in the study. To enhance the representativeness of specific populations of interest in cohort studies, researchers may consider implementing targeted measures that address potential barriers and facilitate their participation. For instance, offering tailored benefits to these populations can be beneficial, including in‐kind benefits such as babysitting or childcare services, reimbursing transportation expenses through vouchers or cash, or coordinating with transportation providers to arrange convenient prepaid services. By proactively addressing the unique needs and challenges these populations face, cohort studies can promote inclusivity and ensure their meaningful involvement, thereby enriching the overall validity and generalizability of the research findings.

Another way of improving the representativeness of cohort studies is implementing a multi‐site approach, which enables a more comprehensive and diverse pool of participants. Establishing enrollment facilities at different levels of the healthcare pyramid, including primary care clinics, community health centers, and tertiary hospitals, increases the likelihood of capturing a broader spectrum of patients, including those with varying degrees of severity. This ensures that even the most severe cases are accounted for and included in the study, thereby avoiding potential bias toward milder cases that may be more easily accessible. While this study only had one community outpatient hospital, other studies may wish to include a mix of both inpatient and outpatient facilities.

Recruiting a reliable and representative cohort of dengue patients is of utmost importance. Therefore, it is critical to capture patients with a broad spectrum of clinical presentations of dengue in the cohort. Characterizing and registering patient (host) characteristics, such as age, gender, occupation, and residence, along with the reasons for testing and outcomes, provide an invaluable resource for clinicians and researchers. Furthermore, it supports the identification of potential risk factors, biomarkers, and social determinants associated with dengue susceptibility, severity, or clinical outcomes. Laboratory testing also aids in understanding the incidence of dengue within and outside the formal healthcare system and patient healthcare‐seeking behavior. The differentiation of DENV serotypes (DENV‐1, DENV‐2, DENV‐3, DENV‐4) is critical for comprehending the epidemiology, virulence, and potential severity associated with each serotype and any potential associations with host characteristics. Last, laboratory tests, such as complete blood count (CBC) and liver function tests, help evaluate disease severity and monitor complications associated with dengue infection, such as plasma leakage or liver dysfunction. Testing in a cohort study of dengue contributes to a comprehensive understanding of dengue epidemiology, pathogenesis, and informs the development of effective preventive and therapeutic strategies.

Our results also showed that the 0–19‐year age group had a higher dengue incidence than the rest of the population. These findings are consistent with the epidemiology of dengue in Thailand, where historically, the younger population has shown higher dengue incidence rates.^7^, ^9^ However, recent systematic reviews of the epidemiology of dengue from 2000 through 2018 suggest that the highest incidence groups have moved from individuals aged 5–14 years in 2000–2011 to those aged 15–24 years in 2011–2018.^7^, ^9^ Data on dengue incidence by age at the subnational level were not publicly available at the time of this manuscript's writing. However, future studies would benefit from comparing dengue incidence to local surveillance data, where available, to better assess the representativeness of their sample.

Our study has some limitations. The study was limited to one district hospital in a mountainous part of Thailand, so our findings may not necessarily be representative of dengue patterns in other regions or hospitals and may not be generalizable to other settings with different geographic or demographic characteristics. Furthermore, the variables included in our study were limited and did not include important factors that may have affected enrollment, such as distance from the hospital, severity of symptoms, and patients' attitudes toward research and their motives to enroll in a health cohort. Understanding participants' motives to enroll in longitudinal health research is a critical factor in the success of these studies.^32^, ^33^, ^34^, ^35^ Participants' willingness to participate in longitudinal health research may be affected by altruism, trust, and personal benefits such as contributing to scientific knowledge, improving healthcare for themselves and others, and benefiting from the research results. Additional variables, such as socioeconomic status, education, and comorbidities, may have provided further insights into the factors that influence dengue incidence and persistence of symptoms and may be relevant factors in determining an individual's willingness to participate.

We can learn from our experience trying to implement a study during the COVID‐19 pandemic, which is a poignant example of the unexpected challenges that can arise. While other disease outbreaks can present significant obstacles, political and security concerns can also create deviations from study plans. To mitigate these risks, researchers and sponsors should anticipate potential challenges and plan how to respond to them. Other aspects of research, including administrative agreements between research funders, institutions, and study sites, can also cause delays. Therefore, careful planning and communication with relevant authorities are essential to ensure the successful execution of future cohort studies.

## 5 CONCLUSIONS

It is helpful to consider how a study's findings extend beyond the specific district, country, time, and disease focus. First, the diagnostic expertise of clinicians is crucial in identifying relevant cases, particularly for conditions that are frequently encountered or have well‐established diagnostic procedures. Conversely, primary care clinicians would likely have limited ability to recognize conditions they rarely encounter. The high rate of laboratory confirmation among clinically diagnosed dengue patients in this study reflects the proficiency of Thai clinicians in interpreting clinical signs and conducting routine lab tests for this endemic condition.

Second, the study highlights the risk of dengue illness across all age groups, with a higher susceptibility observed among younger individuals (0–19 years). This finding underscores the importance of comparing the enrolled cohort to relevant external data sets. While national surveillance data are useful, provincial or district surveillance data would have been better for addressing the cohort's representativeness. Additional data sources, including surveillance data, disease registries, insurance systems, electronic medical records, and studies in related settings, can provide valuable reference data. These studies can also help identify disparities in treatment for the target condition.

Third, the study emphasizes the need to assess underrepresentation of certain groups in cohort studies and take measures to mitigate observed imbalances. In this study, adult females were underrepresented due to childcare and household responsibilities, which hindered their participation in follow‐up interviews at the hospital. To improve cohort representativeness, targeted outreach and recruitment strategies can be implemented to sites or organizations that attract adult females, can be implemented. Less burdensome follow‐up procedures can be developed, and statistical weighting techniques can be employed to increase the importance of underrepresented groups. However, estimates derived from weighted cohorts may have lower precision compared to representative samples. By addressing these considerations, future cohort studies can generate more robust findings, enhance our understanding of the epidemiology of dengue and other target conditions, and guide targeted prevention and management approaches.

## AUTHOR CONTRIBUTIONS

**Donald S Shepard**: Conceptualization; Formal analysis; Methodology; Project administration; Resources; Supervision; Writing—review & editing. **Priya Agarwal‐Harding**: Data curation; Formal analysis; Project administration; Supervision; Writing—review & editing. **Sukhum Jiamton**: Data curation; Software; Writing—review & editing. **Eduardo A Undurraga**: Conceptualization; Formal analysis; Investigation; Methodology; Visualization; Writing—original draft. **Sukhontha Kongsin**: Conceptualization; Data curation; Methodology; Project administration; Supervision; Writing—review & editing.

## CONFLICTS OF INTEREST STATEMENT

All authors received support for this study under a grant from Takeda Vaccines, Inc. to Brandeis University. The funders of this study had no role in the study design, in the collection, analysis, and interpretation of data, in the writing of this manuscript, or in the decision to submit the paper for publication. The authors declare no other conflicts.

## TRANSPARENCY STATEMENT

The lead author Donald S. Shepard affirms that this manuscript is an honest, accurate, and transparent account of the study being reported; that no important aspects of the study have been omitted; and that any discrepancies from the study as planned (and, if relevant, registered) have been explained.

## Supporting information

Supporting information.

## Data Availability

Tabulated data are available through the multiple tables and figures in the main manuscript and supplementary information. Due to privacy commitments to participants, however, person‐level data cannot be made available.
