# Supplementary material for: Enrollment of dengue patients in a prospective cohort study in Umphang District, Thailand, during the COVID‐19 pandemic: Implications for research and policy
Source: Health Sci Rep. 2023 Nov 8;6(11):e1657. doi: 10.1002/hsr2.1657 (PMC10630743; doi:10.1002/hsr2.1657)
Supplement: Supplementary file 1 — Supporting information. [file HSR2-6-e1657-s001.pdf]

Enrolling dengue patients in a prospective cohort study in Umphang District, Thailand: implications for research and policy

Donald S. Shepard, Priya Agarwal-Harding, Sukhum Jiamton, Eduardo A. Undurraga, Sukhonta Kongsin

Supplemental Sections, Tables, and Figures

Contents

Section S1. Dengue epidemiology in Thailand 2

Section S2. Patient inclusion and exclusion criteria 2

Table

Table S1. Cross tabulation of laboratory positivity of dengue cases by NS-1, IgM, and IgG (N=150) 3

Figures

Figure S1. Patient enrollment and compensation process 4

Figure S2. Frequency of patients screened and lab-confirmed recent dengue infection (bi-monthly, December 2020 to November 2021) with % laboratory-confirmed dengue 5

Figure S3. Percent of patients clinically diagnosed with dengue who were laboratory confirmed (NS-1, IgM, either test, both tests) 5

Figure S4. Number of patients screened and lab confirmed by days elapsed since the onset of illness. 6

Figure S5. Number of men and women screened (n=150) and laboratory-confirmed dengue (n=117) patients. 7

References 8

Section S1. Dengue epidemiology in Thailand

The first dengue cases in Thailand were reported in 1949, and all serotypes have circulated since the early 1960s [1-3]. Dengue is now considered a major public health threat in Thailand and is a notifiable disease [1, 2]. Dengue surveillance is passive and records cases, severity, and demographic characteristics of suspected dengue cases. Limited access to healthcare, poverty, misdiagnosis, and limitations in surveillance result in an underestimation of the disease burden of dengue in Thailand [2, 4, 5].

As in other countries, epidemics follow a cyclic pattern and are affected by climate variables, such as rainfall and temperature, with transmission peaks during the wet season (mid-May to mid-October) [2, 6, 7]. The annual incidence of reported dengue ranged from 36 to 137 per 100,000 population for dengue fever and 27 to 101 per 100,000 population for dengue hemorrhagic fever in 2011-2018 [2]. There has been a slow shift in the age group with the highest incidence of dengue from younger individuals aged 5-14 in 2000-2011 moving towards higher prevalence among older individuals aged 15 to 24 years in 2011-2018 [1, 2]. Dengue hemorrhagic fever and dengue shock syndrome were more common among individuals aged 5-14; the more severe cases suggest these children may have a second DENV infection [4]. The overall hospitalization rate was 63% (62-66%) in 2011-2018. [3] Dengue epidemiology varies substantially within the country [1, 2].

There was a total of 81 reported dengue cases in Umphang District from January through December 2020, of which 51 (63%) were classified as dengue fever, and 30 (37%) were classified as dengue hemorrhagic fever. These represent about 11% of the dengue cases reported in Tak Province in 2020 [3].

Section S2. Patient inclusion and exclusion criteria

Eligibility into the study required satisfying all five of these inclusion criteria:

1. The patient has confirmed dengue according to the treating clinician using Thai criteria of clinical diagnosis and routine laboratory testing as well as the additional NS-1 or IgG/IgM.
2. The patient expects to remain in the study area for at least 30 days to be available for in-person follow-up visits.
3. The patient intends to be available to provide follow-up information over the next six months, if needed, either in person or by phone.

4. The patient (if an adult) or caretaker (if a child) understands and can respond to normal questions.

5. The patient (if an adult) or caretaker (if a child) gives appropriate informed written consent and assent for children aged 15-17 years.

The exclusion criteria are failing to satisfy one or more of the above inclusion conditions.

Table S1. Cross tabulation of laboratory positivity of dengue cases by NS-1, IgM, and IgG (N=150)+

<table>
<tr><th colspan="2" rowspan="2"></th><th rowspan="2">IgM lab confirmed</th><th colspan="3">IgG lab positive</th><th rowspan="2">IgG % positive</th></tr>
<tr><th>No</th><th>Yes</th><th>Total</th></tr>
<tr><td rowspan="6">NS1 lab confirmed</td><td rowspan="2">No</td><td>No</td><td>12</td><td>21</td><td>33</td><td>64%</td></tr>
<tr><td>Yes</td><td>14</td><td>25</td><td>39</td><td>64%</td></tr>
<tr><td colspan="2">Subtotal</td><td>26</td><td>46</td><td>72</td><td>64%</td></tr>
<tr><td rowspan="2">Yes</td><td>No</td><td>29</td><td>19</td><td>48</td><td>40%</td></tr>
<tr><td>Yes</td><td>5</td><td>25</td><td>30</td><td>83%</td></tr>
<tr><td colspan="2">Subtotal</td><td>34</td><td>44</td><td>78</td><td>56%</td></tr>
<tr><td rowspan="4">NS1 and/or IgM confirmed</td><td colspan="2">Both NS1 and IgM negative</td><td>12</td><td>21</td><td>33</td><td>36%</td></tr>
<tr><td colspan="2">NS1 and/or IgM positive</td><td>48</td><td>69</td><td>117</td><td>59%</td></tr>
<tr><td colspan="2">Total screened</td><td>60</td><td>90</td><td>150</td><td>60%</td></tr>
<tr><td colspan="2">Both NS1 and IgM positive</td><td>5</td><td>25</td><td>30</td><td>83%</td></tr>
<tr><td rowspan="3">IgM lab confirmed</td><td colspan="2">No</td><td>41</td><td>40</td><td>81</td><td>49%</td></tr>
<tr><td colspan="2">Yes</td><td>19</td><td>50</td><td>69</td><td>72%</td></tr>
<tr><td colspan="2">Total screened</td><td>60</td><td>90</td><td>150</td><td>60%</td></tr>
</table>

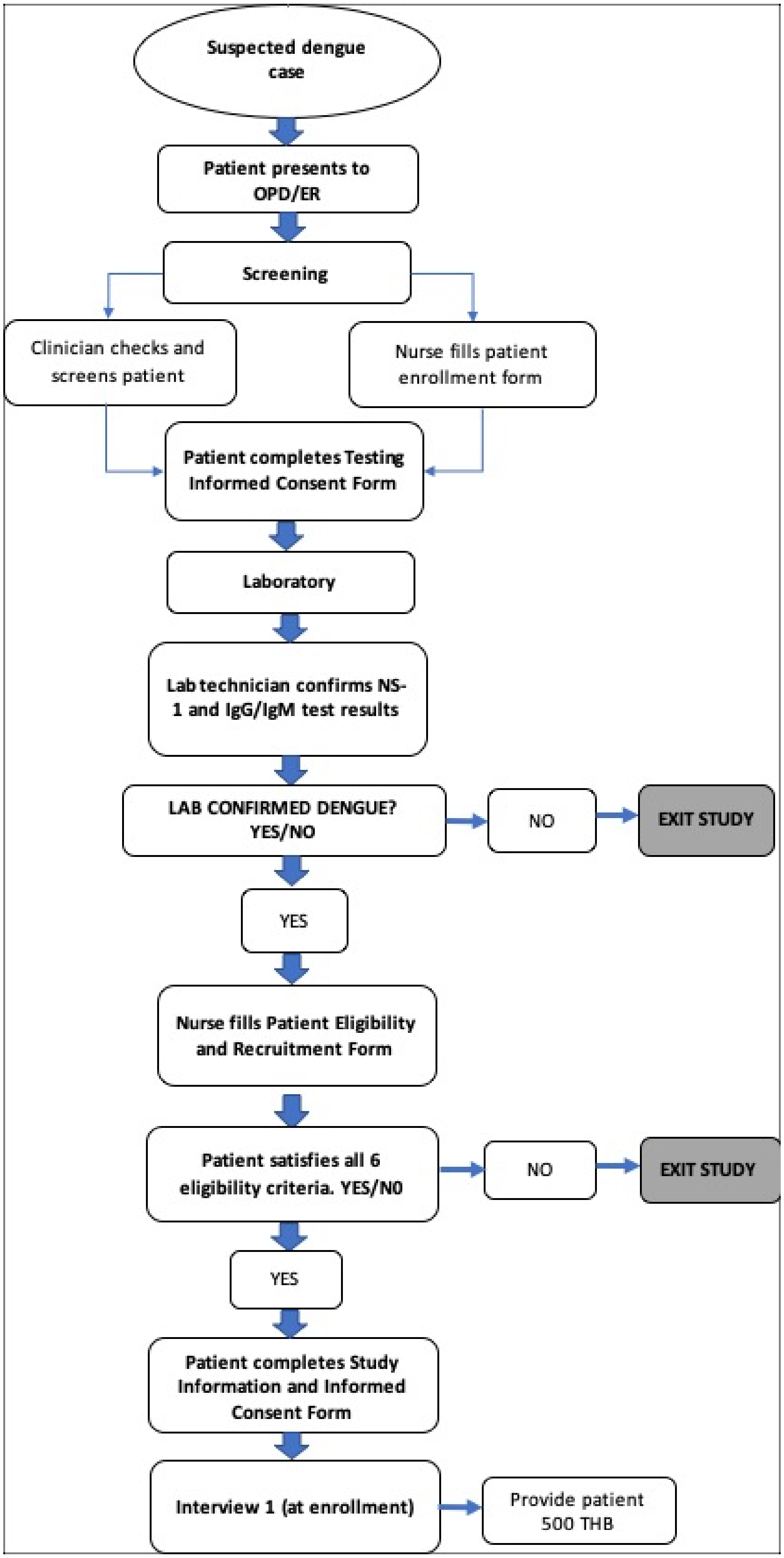

Figure S1. Patient enrollment and compensation process

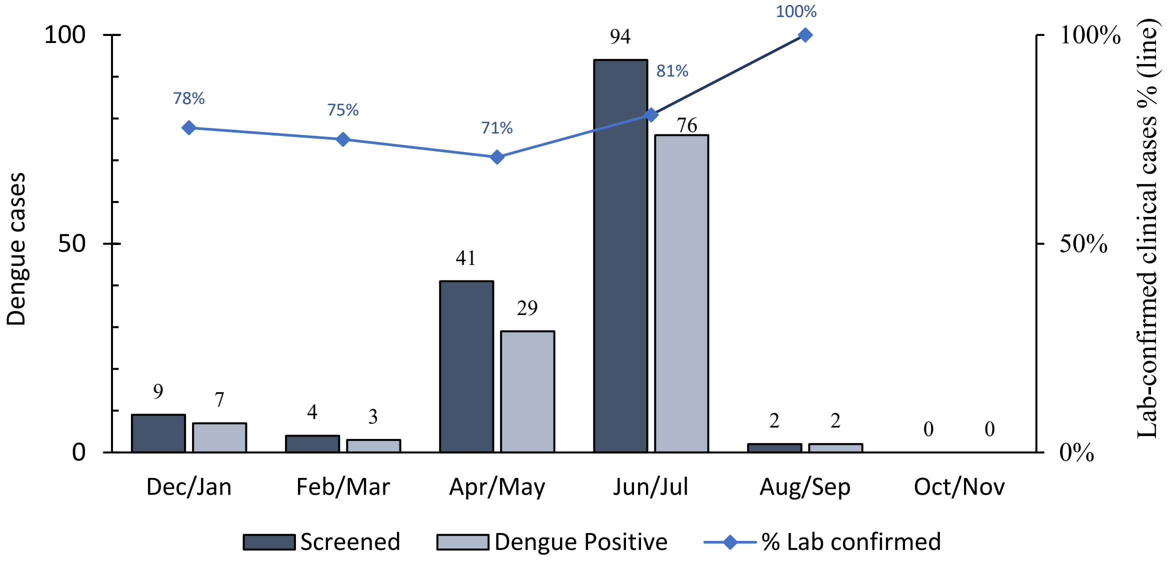

Figure S2. Frequency of patients screened and lab-confirmed recent dengue infection (bi-monthly, December 2020 to November 2021) with % laboratory-confirmed dengue.

Dengue positive was defined as a recent laboratory confirmed DENV infection with NS1 and/or IgM positive.

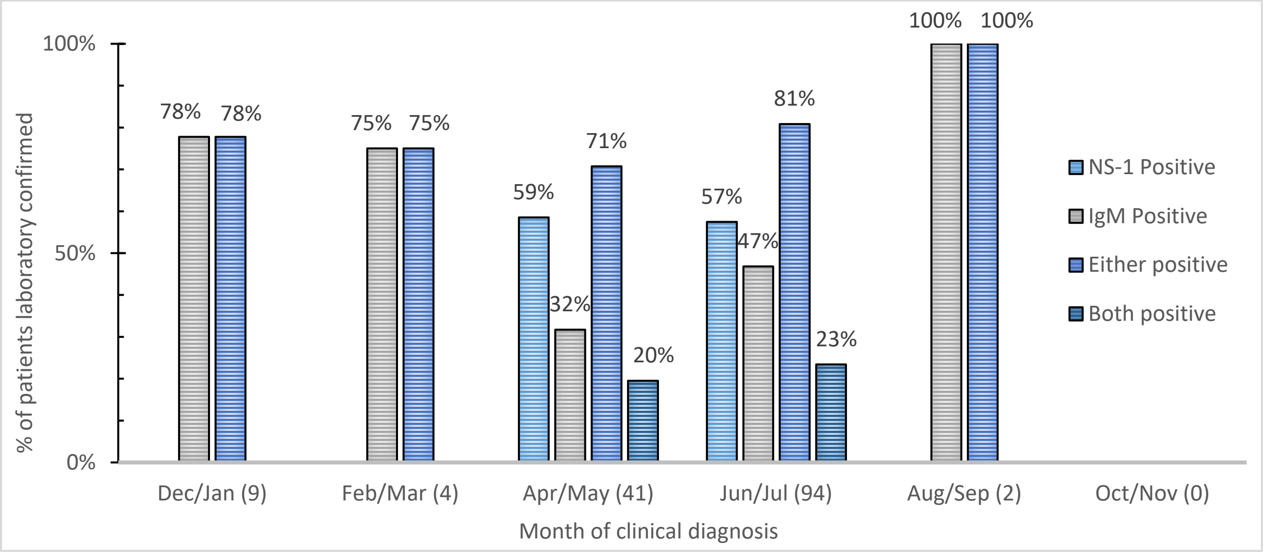

Figure S3. Percent of patients clinically diagnosed with dengue who were laboratory confirmed (NS-1, IgM, either test, both tests)

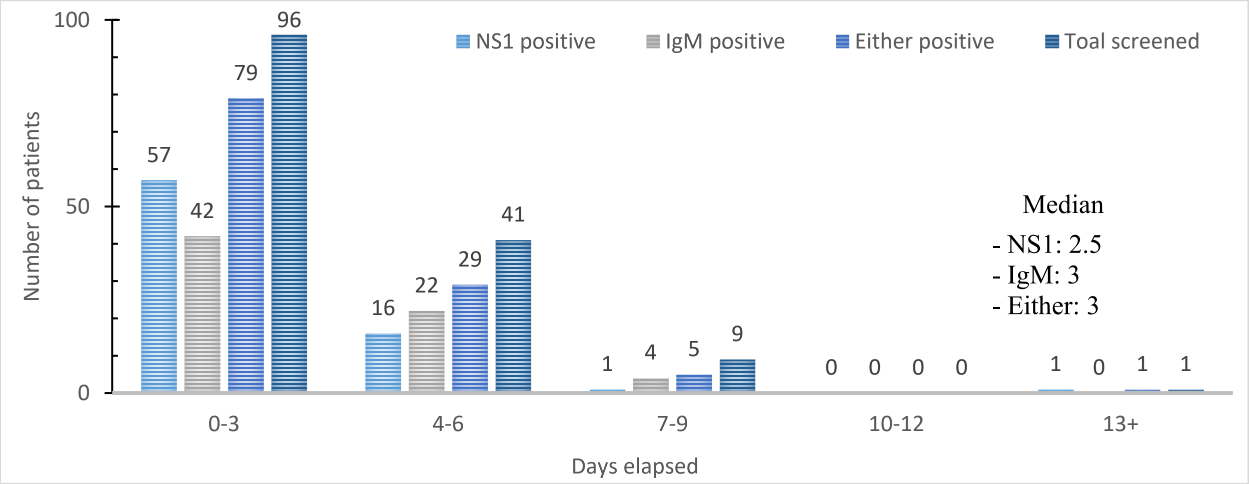

Figure S4. Number of patients screened and lab confirmed by days elapsed since the onset of illness. The onset of illness is self-reported. The total number of symptomatic patients screened was 147 because 3 patients did not report any symptoms before screening and were therefore excluded from this figure. The breakdown of the 147 patients by testing status is 75 NS1 positive, 68 IgM positive, 29 both NS1 and IgM positive, 114 NS1 and/or IgM positive, and 33 neither NS1 nor IgM positive.

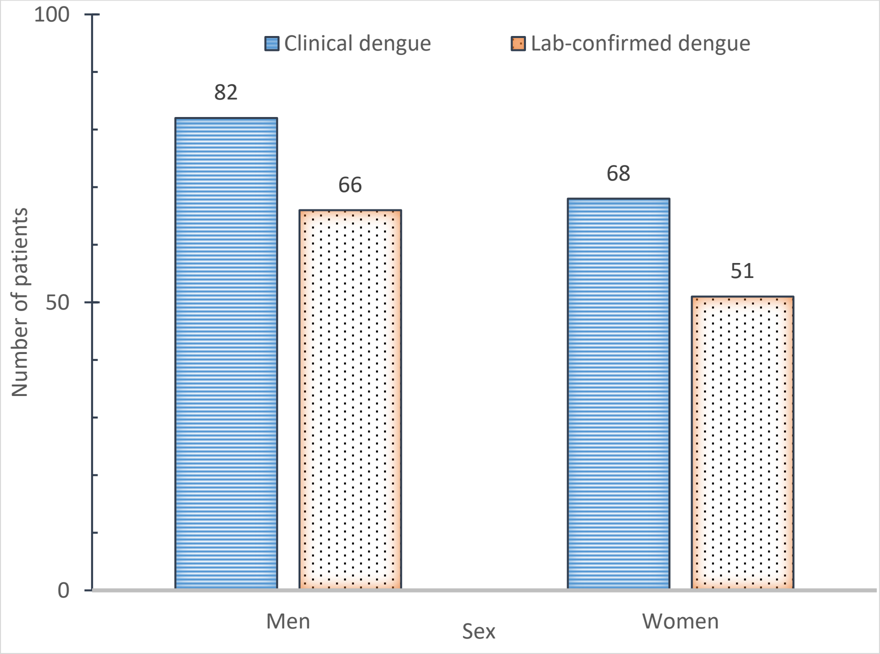

Figure S5. Number of men and women screened regardless of symptoms (n=150) and laboratory-confirmed dengue (n=117) patients.
Dengue positive was defined as a recent laboratory-confirmed DENV infection with NS1 and/or IgM positive. 80% of men, 75% of women, and 78% overall had laboratory confirmation.

References

1. Limkittikul K, Brett J, L'Azou M. Epidemiological trends of dengue disease in Thailand (2000–2011): A systematic literature review. PLoS Neglected Tropical Diseases. 2014;8(11):e3241. doi: 10.1371/journal.pntd.0003241.

2. Thisyakorn U, Saokaew S, Gallagher E, Kastner R, Sruamsiri R, Oliver L, et al. Epidemiology and costs of dengue in Thailand: A systematic literature review. PLOS Neglected Tropical Diseases. 2022;16(12):e0010966.

3. Ministry of Public Health Thailand. District dengue data Bangkok, Thailand: Bureau of Epidemiology - Department of Disease Control; 2022 [cited 2023 January 2]. Available from: http://doe.moph.go.th/surdata/disease.php?ds=26#.

4. Wichmann O, Yoon IK, Vong S, Limkittikul K, Gibbons RV, Mammen MP, et al. Dengue in Thailand and Cambodia: An assessment of the degree of underrecognized disease burden based on reported cases. Plos Neglected Tropical Diseases. 2011;5(3):e996. doi: e99610.1371/journal.pntd.0000996. PubMed PMID: ISI:000288940800027.

5. Undurraga EA, Halasa YA, Shepard DS. Use of expansion factors to estimate the burden of dengue in Southeast Asia: A systematic analysis. PLoS Neglected Tropical Diseases. 2013;7(2):e2056. doi: 10.1371/journal.pntd.0002056.

6. Xu Z, Bambrick H, Yakob L, Devine G, Lu J, Frentiu FD, et al. Spatiotemporal patterns and climatic drivers of severe dengue in Thailand. Science of The Total Environment. 2019;656:889-901.

7. Sabchareon A, Sirivichayakul C, Limkittikul K, Chanthavanich P, Suvannadabba S, Jiwariyavej V, et al. Dengue Infection in Children in Ratchaburi, Thailand: A Cohort Study. I. Epidemiology of Symptomatic Acute Dengue Infection in Children, 2006-2009. Plos Neglected Tropical Diseases. 2012;6(7):e1732. doi: 10.1371/journal.pntd.0001732. PubMed PMID: WOS:000307101900025.
